# Simulation study on collaborative shopping formation from the perspective of group structure and two-dimensional opinion

**DOI:** 10.1016/j.heliyon.2024.e38995

**Published:** 2024-10-05

**Authors:** Shulin Liang, Wang Hu

**Affiliations:** a Management School, Hunan City University, Yiyang, 413000, Hunan, China; b Hunan New-type Urbanization Institute, Yiyang, 413000, Hunan, China

**Keywords:** Collaborative shopping, Group structure, Two-dimensional opinion, Numerical simulation, DW model

## Abstract

Group communication and collaborative shopping models are favoured by consumers, so research on group opinion has attracted the attention of businesspeople. Based on the dual-process model (DW model), interaction rules for developing consumer opinions are constructed, and the influence of factors such as group structure, sponsor characteristics, and sub-opinions on collaborative shopping is investigated via numerical simulations. The results show that the group structure of consumers is closely related to the success of the joint purchase. The sponsor influences the development of the sub-options of the group, which can split the original convergent and consistent opinions, and the sub-options of the group can split within a specific range. The conclusions can provide theoretical support for businesspeople to successfully carry out collaborative shopping and promote the vigorous development of social commerce.

## 1 Introduction

Many people shop together with others. They exchange information via social media before or during their shopping trip [[1], [2], [3], [4]]. Existing research has focused on the influence mechanism of individual consumer purchasing. The influence mechanism relates to the factors and processes that influence the consumers' buying decisions for a product, such as the quality of the product, its price, their desire to use it, and influence by others [[5], [6], [7], [8]]. Existing consumer behaviour models highlight the importance of creating valuable products and services to meet customer needs. According to Blackwell et al. [9], consumer behaviour is the process of purchasing, consuming, and ordering products and services. Schiffman and Kanuk [10] describe consumer behaviour as selecting personal or household products depending on available resources such as money, time, and effort. As defined by Gabbott, Hogg and Blackwell et al. [11,12], consumer behavior includes the activities and processes involved in purchasing and disposing of products or services based on personal experiences and ideas. The usefulness of tailored advertising through membership systems for e-commerce platforms has been studied recently, and the results offer insights into how customized marketing techniques might improve customer engagement and increase sales [56]. Frederick and Salter's [13] theory states that customers are satisfied because of the value of a package, which includes their judgments of price, product quality, service, and corporate image. They further argued that customers would be satisfied if the value package delivered to them fulfilled their expectations. Several theorists, including Rowley, Fredericks, Salter and Blackwell et al. [13,14], have argued that understanding and creating the elements influencing consumer satisfaction requires an awareness of the uniqueness of the product. However, customers must comprehend the good and accept the company's standards to be satisfied. Furthermore, there are two ways to assess consumer behaviour: the decision-making process involved in a purchase and the factors that influence the purchase process. To explore the interaction dynamics behind the development of consumer opinions in collaborative shopping scenarios, this study expands on the dual-process model (DW model) [15,16], drawing on the extensive literature on consumer behaviour. Building on foundational developments in consumer behaviour research [[17], [18], [19]], we create interaction rules that consider sub-opinions, sponsor characteristics, and group structure.

The following are some examples of collaborative shopping in real life. The family of all headings goes to the grocery store. Children come alongside their parents to be required to contribute in one way or another when their parents ask them which snacks they should buy, and they select their favourite snacks [20]. Families specifically for schooling purchase stationery and other items like clothes or shoes. The children decide on the items to buy, and parents ensure that the items are affordable and meet the school's standards [21]. Apartment mates buy groceries, cleaning accessories, and personal effects for the house in one basket. To achieve this, they agree on aspects such as brands, quantities, and budgets to meet all the required requirements [22]. A set of friends goes shopping to purchase a birthday present for one of their friends. They discuss and shop for one gift they are sure the recipient will appreciate [23]. People shopped for home renovation products such as paint, furniture, and decorations in pairs or with other family members. They can also discuss the options and possibilities of models and ensure that what is developed is a favourite to all [24]. Groups of friends or related persons go out shopping for wear, accessories, and vacation items during a shopping spree. They agree to properly acquire all the items and accessories required during the journey. Employees proceed to shop and purchase articles needed for an office party or an official activity. They coordinate what supplies, decorations, or equipment should be bought to ensure a good event occurs [25,26]. People create groups to share and look out for products on Facebook Marketplace or any other buying-selling interface. This will portray the common aspect of shopping; for instance, members may work together to bargain for prices, share pictures and reviews, and even share information on exciting deals. Flash sale sites are those where products are available at lower prices for a limited time, and sometimes, the number of products to be bought to create the sale has to be reached. In turn, people act in unison to provide the required quotas for purchase, which illustrates the integrated approach to obtaining specific offers [27]. Examples of collaborative shopping demonstrate how social interactions and group decision-making affect consumer choices. These real-world examples highlight the usefulness of collaborative shopping concepts and highlight the important influence that social interactions and group dynamics have on consumer behavior.

Despite the growing importance of collaborative buying behavior in the digital era, little is known about it, especially when it comes to group structure and two-dimensional opinion dynamics. Although previous research has recognized the impact of social dynamics and opinion formation on group decision-making processes, few simulation-based studies have explicitly looked at how different group structures affect the emergence and development of cooperative shopping behavior. In addition, further research is necessary to fully comprehend how two-dimensional opinion factors like social influence and personal preference interact to influence group purchase decisions. Recent developments in opinion dynamics modelling and simulation techniques present new potential to close this gap. This highlights the necessity for an extensive study that incorporates these characteristics to give a more nuanced understanding of the establishment of collaborative shopping. If marketers and online platforms want to adjust their strategies to the intricacies of group-based customer behavior, this research could have a big impact on them [[28], [29], [30], [31], [32]].

Incorporating related consumer behaviour theories and models outside of the DW framework would be advantageous in strengthening the study's theoretical contribution. The theory of planned behaviour (TPB) is one such theory that might be combined. According to the TPB, three elements determine behavioural intention: perceived behavioural control, subjective norms, and attitudes toward conduct [33]. The integration of the TPB enhances their understanding of how consumer attitudes, social influences, and perceived control impact their involvement in cooperative shopping. Social identity theory (SIT) is another pertinent theory. According to SIT, people's behaviour is driven by the need to maintain a positive social identity, and their self-concept is derived from their affiliation with a particular social group [34]. SIT investigates how customers' affiliation with certain groups or communities affects their behaviour in collaborative purchasing environments. The Technology Acceptance Model (TAM) may provide valuable insights into customer acceptance and uptake of collaborative shopping platforms. The TAM reveals that perceived utility and ease of use are essential predictors of users' attitudes toward technology adoption [35]. Understanding the factors that influence customer engagement with collaborative shopping platforms can be aided by integrating TAM. Group perspectives and collaborative buying behaviour can be more thoroughly analyzed by incorporating these and other pertinent theories and models into the study's framework. This strategy would improve the study's theoretical contribution and offer insightful information to social commerce practitioners and academics.

This paper uses the DW model to investigate how group opinions change during collaborative shopping, emphasizing interaction dynamics. It builds on previous studies on customer behaviour and suggests interaction guidelines that consider sponsor attributes, group dynamics, and sub-options.

The DW model recognizes the dynamic interactions between people in collaborative shopping scenarios, enabling a sophisticated understanding of the evolution of group opinions. The model considers sponsors' impact and attributes, recognising their diverse functions within organizations with distinct organizational structures. This approach offers a more accurate depiction of the dynamics of collaborative shopping. The DW model thoroughly explains the variables influencing collaborative shopping by accounting for group structure's influence and sub-opinions' development.

However, as a group consumption activity, individuals’ involvement in collaborative shopping is bound to be affected by group factors, and the formation of collaborative shopping also requires the evolution of group opinions to tend to agree [[36], [37], [38]]. Therefore, the evolution trend of group opinions and the critical factors for forming collaborative shopping will be explored. Based on the analysis of the interaction model, the interaction rules between different types of agents are constructed, including a simulation model of one- and two-dimensional opinions, and through computer experiments, the evolution process of group opinions and sub-opinions is simulated to explore the evolution trend and critical factors.

## 2 Agents and their attributes

In a collaborative shopping platform, the sponsor publicly publishes product information, participation rules, payment methods, etc. Interested users communicate and interact with sponsors and other users [[39], [40], [41]]. As a few unique users, opinion leaders play a specific guiding role in users' psychology and behaviour [[42], [43], [44]]. Therefore, this paper divides collaborative shopping participants into three main categories: sponsors, opinion leaders, and consumers. The sponsor attributes are set as opinion A_k_, opinion quality B_k,_ professionalism C_k,_ and interactivity D_k_, and the corresponding values are A_k_ ∈ [0.8, 1], B_k_ ∈ [0.8, 1], C_k_ ∈ [0, 1] and D_k_ ∈ [0,1]. Similarly, consumers' attributes are set as opinion E_j_(t), opinion quality F_j_, conformity G_j,_ and trust tendency H_j_, all of which are between 0 and 1. Opinion leaders’ attributes are set as opinion I_h_, opinion quality J_h_, activity K_h_ and cohesion L_h_, and the values of the attributes are I_h_ ∈ [0,0.2] or [0.8,1], J_h_ ∈ [0.8,1], K_h_ ∈ [0.8,1], and L_h_ ∈ [0.8,1], respectively.

## 3 Interaction rules

The particular modifications and guidelines address the shortcomings of the original model, such as its one-dimensional opinion scope and oversimplified interaction rules, and are therefore required to increase the DW model's applicability to collaborative shopping scenarios. The model's ability to reflect the intricacies of group dynamics and individual influences in collaborative shopping scenarios is enhanced by the addition of variable convergence coefficients, optimization of interaction circumstances, and integration of multidimensional opinions. Consequently, opinion evolution in these settings becomes more precisely and nuancedly understood [[45], [46], [47]].

### 3.1 DW model and its improvement strategy

The DW model proposed by Deffaunt et al. defines an individual opinion as a random number in a continuous interval [48]. In the model, individuals i and j are randomly selected; if the difference in opinions between them is less than a certain threshold ε, namely∣x_i_-x_j_∣﹤ε, individuals i and j communicate their opinions, or they do not [49]. The interaction rules are as follows:

x_i_(t+1) = x_i_(t)+ μ(x_j_(t)- x_i_(t))

x_j_(t+1) = x_j_(t)+ μ(x_i_(t)- x_j_(t))

The DW model reveals the evolution of group opinions at the macro level through reasonable assumptions of individuals’ interaction behaviours at the micro level. However, the interaction rules of the DW model seem simple and rough, which makes it difficult to accurately describe the evolution process of group opinions in collaborative shopping. Therefore, it is necessary to improve the DW model according to the characteristics of group consumption to enhance the explanatory power for the interaction of consumer groups. The DW model can be improved in the following ways [50]:

First, the convergence coefficient μ increases. In the DW model, the convergence coefficient μ reflects the acceptance of others' opinions, and differences in individuals make the acceptance of others' views different; therefore, a constant μ cannot truly reflect the differences among individuals in a group, and μ can be set as a variable obeying a specific law to reflect the interaction of individuals’ opinions truly. Therefore, the convergence coefficient μ is improved into an influence function, and a function of the influencing factors of individual pinions is established.

Second, the interaction conditions were optimised. In the DW model, the threshold ε restricts the interaction objects. The more comprehensive, objective, and vivid the information, the more easily individual consumers adopt opinions; therefore, when individuals with opinion differences are within the threshold ε interact, an individual with more abundant and convincing information can influence the opponent to modify his opinion. Accordingly, individual opinions with low-quality change, while those with high quality remain constant [51].

Third, for the threshold value ε, online social networks gather many users based on familiar topics or interpersonal relationships, forming different groups [52]. Some group members have high similarity, while others have significant individual differences; therefore, the threshold ε should be set differently according to group nature. The threshold ε can be fixed for the homogeneous group and set to follow a specific distribution for group users with significant differences.

Fourth, there has been a breakthrough in the limitations of one-dimensional opinion. The opinion of consumers participating in collaborative shopping is set as any continuous value on the interval [0,1]. The setting synthesises consumers’ thoughts; however, the diversity of collaborative shopping determines that consumer opinion comprises many aspects, such as the product, price, time, and reference group. Therefore, the opinion can be decomposed; accordingly, a simulation model of multidimensional opinion can be established.

### 3.2 Interaction rules of one-dimensional opinion

(1) Interaction rules between sponsor and individual consumers [53].

If B_k_ > F_j_

E_j_(t+1) = E_j_(t)+(A_k_-E_j_(t))r(k,j)

If B_k_ ≤ F_j_

E_j_(t+1) = E_j_(t)

(2) Interaction rules between opinion leaders and individual consumers

If| I_h_-E_j_(t)|＞ε

E_j_(t+1) = E_j_(t)

If| I_h_-E_j_(t)|<ε, and J_h_ < F_j_

E_j_(t+1) = E_j_(t)

If| I_h_-E_j_(t)|<ε, and J_h_(t) > F_j_

E_j_(t+1) = E_j_(t)+(I_h_-E_j_(t))s(h,j)

(3) Interaction rules between individual consumers

If| E_i_(t)- E_j_(t)|<ε, and F_i_> F_j_

E_j_(t+1) = E_j_(t)+(E_i_(t)-E_j_(t))w(i,j)

E_i_(t+1) = E_i_(t)

If| E_i_(t)- E_j_(t)|<ε, and F_j_>F_i_

E_i_(t+1) = E_i_(t)+(E_j_(t)-E_i_(t))w(j,i)

E_j_(t+1) = E_j_(t)

### 3.3 Interaction rules of two-dimensional opinion

Since collaborative shopping opinions are multidimensional, comprehensive opinions can be further decomposed into a multidimensional opinion matrix to discuss the evolution of group opinions [54].

First, individual consumer j holds an F-dimensional opinion, which the following vector can represent:

x_j_ = [ x_j1,_ x_j2, …_x_jF_], x_jf_ ∈ [0,1], f = 1,2 … F

Second, according to the interaction condition of the DW model, only individuals whose opinions differ within the threshold range can interact. When an individual consumer has a multidimensional opinion, the Euclidean distance can describe the multifaceted opinion difference between two consumers. The Euclidean distance is a frequently used definition of distance. It refers to the distance between two points in the m-dimensional space or the natural length of a vector (that is, the distance from the point to the original point). According to the Euclidean distance, the difference in opinions between two individuals with multidimensional views can be expressed as follows:

$$\varepsilon =\sqrt{\sum_{f=1}^{F}{\left(x_{if}-x_{if}\right)}^{2}}$$

Accordingly, the DW model from the multidimensional perspective can be improved as follows:

x_if_(t+1) = x_if_(t)+ μ(x_jf_(t)- x_if_(t))

x_jf_(t+1) = x_jf_(t)+ μ(x_if_(t)- x_jf_(t))

f = 1,2 … F

To gain a deeper understanding of the evolution law from a multidimensional perspective, this paper chooses a two-dimensional perspective to explore and establish the interaction rules between agents from a two-dimensional perspective as follows:

(1) Interaction rules between sponsor and individual consumers

If B_k_ > F_j_

E_j1_ (t+1) = E_j1_(t)+(A_k1_-E_j1_(t))r(k,j)

E_j2_ (t+1) = E_j2_(t) + (A_k2_ - E_j2_ (t)) r(k,j)

If B_k_ ≤ F_j_

E_j1_(t+1) = E_j1_(t)

E_j2_(t+1) = E_j2_(t)

(2) Interaction rules between opinion leaders and individual consumers

If ≥ε

E_j1_(t+1) = E_j1_(t)

E_j2_(t+1) = E_j2_(t)

$$\sqrt{{\left(I_{h1}\left(t\right)-E_{j1}\left(t\right)\right)}^{2}+{\left(I_{h2}\left(t\right)-E_{j2}\left(t\right)\right)}^{2}}$$

If﹤ε and J_h_ ≤ F_j_

E_j1_(t+1) = E_j1_(t)

E_j2_(t+1) = E_j2_(t)

If﹤ε; and J_h_ > F_j_

E_j1_(t+1) = E_j1_(t)+(I_h1_-E_j1_(t))s(h,j)

E_j2_(t+1) = E_j2_(t)+(I_h2_-E_j2_(t))s(h,j)

$$\sqrt{{\left(E_{h1}\left(t\right)-E_{j1}\left(t\right)\right)}^{2}+{\left(E_{h2}\left(t\right)-E_{j2}\left(t\right)\right)}^{2}}$$

(3) Interaction rules between individual consumers

If ﹤ε; and F_i_ > F_j_

$$\sqrt{{\left(E_{h1}\left(t\right)-E_{j1}\left(t\right)\right)}^{2}+{\left(E_{h2}\left(t\right)-E_{j2}\left(t\right)\right)}^{2}}$$

E_j1_(t+1) = E_j1_(t)+(E_i1_(t)-E_j1_(t))w(i,j)

E_j2_(t+1) = E_j2_(t)+(E_i2_(t)-E_j2_(t))w(i,j)

E_i1_(t+1) = E_i1_(t)

E_i2_(t+1) = E_i2_(t)

If<ε and F_i_ < F_j_

E_i1_(t+1) = E_i1_(t)+(E_j1_(t)-E_i1_(t))w(j,i)

E_i2_(t+1) = E_i2_(t)+(E_j2_(t)-E_i2_(t))w(j,i)

E_j1_(t+1) = E_j1_(t)

E_j2_(t+1) = E_j2_(t)

B_k_, F_j_, and I_h_ denote the sponsors', customers', and opinion leaders' respective levels of opinion quality. A_k_ and J_h_ stand for opinion leaders' and sponsors' professionalism and cohesiveness. Sponsors' and opinion leaders' cohesion, activity, and interaction are represented by the following attributes: C_k_, D_k_, K_h_, and L _h_ represent the changing views of specific customers in one- and two-dimensional spaces using the variables Ej(t), E_j1_(t), E _j2_(t), Ei(t), E_i1_(t), and E_i2_(t), respectively. The influence functions r(k,j), s(h,j), and w(i,j) assess the impact of sponsors, leaders of opinion, and individual customers on one another in light of several variables, such as conformity, cohesion, opinion quality, and interactivity. Fj depicts the inclination of individual consumers toward conformity or trust.

### 3.4 Establishment of the influence function

In the DW model, the convergence coefficient μ represents the individual acceptance of others’ opinions in the interaction process. The acceptance degree of individual consumers is limited by information content, personality, other situations, and so on; therefore, the convergence coefficient μ is improved to establish the influence function, and the analytic hierarchy process is adopted to determine the weight and test the consistency. The results are as follows:

Influence function of sponsor impact on individual consumers

r(k,j) = 0.187C_k_+0.326D_k_+0.215G_j_+0.163H_j_+0.109 (1- F_j_)

Influence function of opinion leaders impacting individual consumers

s(h,j) = 0.256K_h_+0.350L_h_+0.166G_j_+0.110H_j_+0.118 (1- F_j_)

Influence function between individual consumers

W (I, j) = 0.349 G_j_+ 0.484 H._j_+ 0.167 (1 - F_j_)

## 4 Simulation experiment

### 4.1 Experimental design

The group size of collaborative shopping is set as 150 [55]. In the study of homogeneous thresholds, it is often believed that 0.25 can genuinely reflect the situation of a real society; therefore, the homogeneous threshold is set to 0.25 in the simulation experiment, and the heterogeneous threshold ε follows a random uniform distribution. In addition, with the help of simulation experiments, the influence of changes in the ratio of opinion leaders is explored to determine the number of opinion leaders. The results show that opinion leaders play a differentiating role in group opinion evolution, and the greater the ratio of opinion leaders is, the stronger the differentiation of group opinions. Still, this influence gradually disappears when the ratio of opinion leaders reaches a specific value. The median value of 12 is taken as the number of opinion leaders for the study. In the simulation experiment, the ordinate represents the opinion value of the collaborative shopping group, and the abscissa represents the simulation steps. This study utilized Matlab for numerical simulation.

### 4.2 Evolution trend of group opinions with different structures

**Fig. 1:**
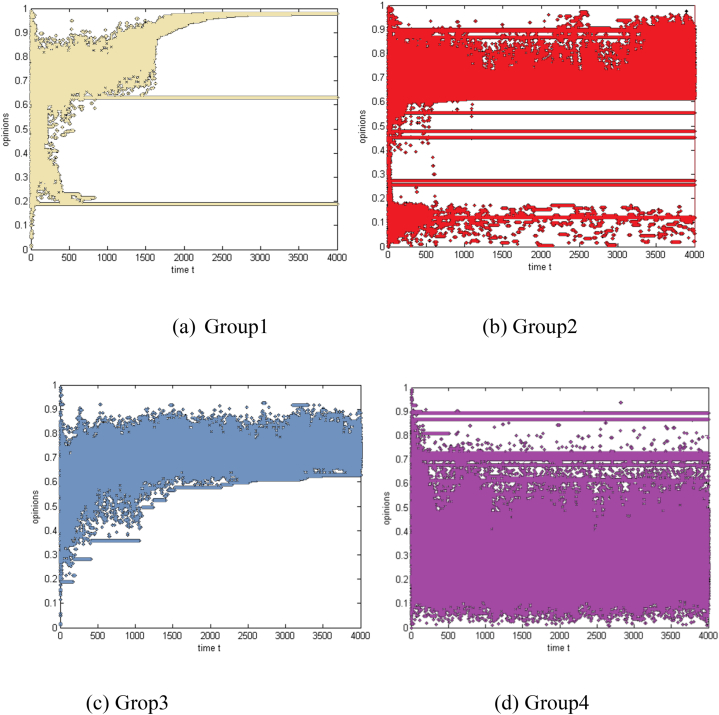
The evolution of group opinions with different structures (a) Homogeneous threshold and simple hierarchy (Group 1) (b) Homogeneous threshold and complex hierarchy (Group 2) (c) Heterogeneous threshold and simple hierarchy (Group 3) (d) Heterogeneous threshold and complex hierarchy (Group 4).

As a result of group hierarchy and individual differences, based on the agent categories and the nature of the threshold in the group, collaborative shopping is divided into four categories. The opinion evolution laws of every category are analyzed to confirm the most suitable category appropriate for carrying out collaborative shopping and beneficial for improving the success rate of activities. To compare the evolution laws of group opinions with different structures more clearly and intuitively, the figures are summarized as follows:

A basic hierarchy is used to illustrate the homogeneous threshold in Figure (1a). Every customer has an identical threshold value, and there is a simple group hierarchy free of distinctions. A homogeneous threshold with a complex hierarchy is depicted in Figure (1b). Although the group has a clear hierarchical structure that may be impacted by opinion leaders, consumers have a comparable threshold value. A heterogeneous threshold with a straightforward hierarchy is depicted in Figure (1c) Customers' thresholds fluctuate according to a random distribution, and there is little hierarchy and a simple group structure. A heterogeneous threshold with a complex hierarchy is shown in Figure (1d) Customers' thresholds differ, and there is a clear hierarchical structure within the group that includes opinion leaders' impact.

The simulation results show that group 3 will likely succeed in collaborative shopping. All consumers in group 3 agree with the activity, but the degree of agreement differs. The possible reasons are as follows: a heterogeneous threshold means differences between consumers, which promotes more communication and helps group members achieve consensus. Furthermore, without the differentiation effect of opinion leaders, sponsors possessing firm opinions and detailed opinion quality push moderate views in a more positive direction.

The groups that could carry out collaborative shopping successfully included Group 1 and Group 2. Although there are still many opponents, most consumers hold positive opinions. The possible reason is that in Group 1, the immobilised threshold prompts some consumers with similar opposition to gather; accordingly, the values of opposed opinions converge. Moreover, there is no differentiation effect of opinion leaders, so the sponsor pushes parts of the consumer on the two sides to have more favourable opinions, which even mutate and polarise after some time. However, in Group 2, opinion leaders intensify further differentiation of group opinions. Still, the sponsor reverses some objectors' opinions and converges the approvers' opinions at a specific interval. That sponsor reverses some opposing opinions and converges positive opinions, promoting the possibility of successful progress.

Group 4 is the most unlikely group to carry out collaborative shopping successfully. The heterogeneous threshold promotes more communication and dispersion of opinions, which further aggravates the participation of opinion leaders, so group opinions are in a rupturing condition. The sponsor only positively affects a few consumers who cannot change the situation of numerous opponents.

### 4.3 The role of the sponsor

To explore the role of the sponsor in group 3, the characteristic parameters of the sponsor are adjusted. The values of professionalism are set as [0, 0.2] and [0.8, 1], the values of interactivity are set as [0, 0.2] and [0.8,1], and the characteristics of the sponsor are set as [0,0.2] and [0.8,1]. Then, the evolution of group opinions is compared. In group 3, the opinions are always greater than 0.5; therefore, the average opinion is calculated to compare the influence of different characteristics.

**Fig. 2:**
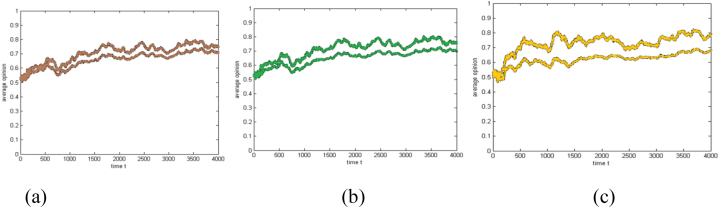
The evolution of the average opinion at different levels of sponsor characteristics (a) Professionalism comparison (b) Interactivity comparison (c) Synchronous comparison of the two characteristics.

Figure (2a) shows how the group's average opinions have changed between times when the sponsor's professionalism has increased and decreased. The progression of the group's average attitudes at low and high degrees of sponsor interaction is compared in Figure (2b). The evolution of average opinions at low and high degrees of professionalism and interactivity is examined in Figure (2c).

The simulation experiments show that the average opinion of the group rises steadily at first and then fluctuates within a specific range for both high-level and low-level sponsor characteristics. At T = 4000, when the professionalism or interactivity of the sponsor is at an interval from 0 to 0.2, the average group opinion fluctuates at approximately 0.7; when the professionalism or interactivity of the sponsor is at an interval from 0.8 to 1, the average group opinion fluctuates at approximately 0.75; when the professionalism and interactivity of the sponsor are both at a low level from 0 to 0.2, the average group opinion is always close to but not higher than 0.7; when the professionalism and interactivity of the sponsor are both at a high level from 0.8 to 1, the average group opinion fluctuates at approximately 0.8.

### 4.4 The evolution of group sub opinions

(1) The evolution of group sub-opinions without a sponsor

**Fig. 3:**
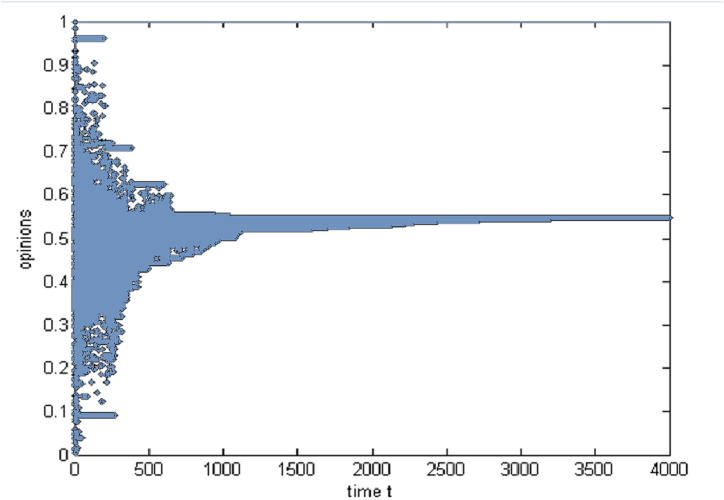
Group sub-opinion evolution from the perspective of two-dimensional opinion.

A simulation experiment on the interaction between individual consumers is first conducted to explore the evolution laws of group sub-opinions, which provides a foundation for subsequent comparative experiments. Assuming 150 consumers, each consumer has two subopinions, and the initial values of the two subopinions follow a random uniform distribution. The ordinate represents the group subopinions, and the abscissa represents the simulation steps. The threshold ε is set to homogeneity at different levels (1.4, 1, 0.5, 0.25, and 0.1) and to a heterogeneous threshold of random uniform distribution. Simulation experiments are carried out according to the interaction rules of two-dimensional opinions, and representative results are shown in Fig. 3.

Figure (3) shows how the convergence of group sub-opinions over simulation steps is influenced by varying degrees of heterogeneous and homogeneous thresholds. The simulation results show that the group sub-opinions converge to 0.55 in the case of the heterogeneous threshold. When the threshold is homogeneous, there are slight differences in the evolution results at different levels, but in the nonextreme case, the group sub-opinions converge to approximately 0.55. The evolution results of the group sub-opinions are not affected by a threshold almost the same as the evolution trend of the one-dimensional opinion in the same type of group. However, the convergence of the group subopinions is faster, and the system needs less time to reach stability.

(2) The addition of sponsor

The above simulation experiments prove that the threshold does not affect the evolution of group sub-opinions. Considering the natural differences among individuals in a group, the threshold set for heterogeneity is closer to reality. In the subsequent simulation experiments, all thresholds follow a random uniform distribution. In addition, group 3 is most likely to succeed in collaborative shopping in the simulation experiments of one-dimensional opinions, while group 4 is unsuitable. Therefore, the following section for comparative analysis discusses the evolution trend of group sub-opinions in group 3.

**Fig. 4:**
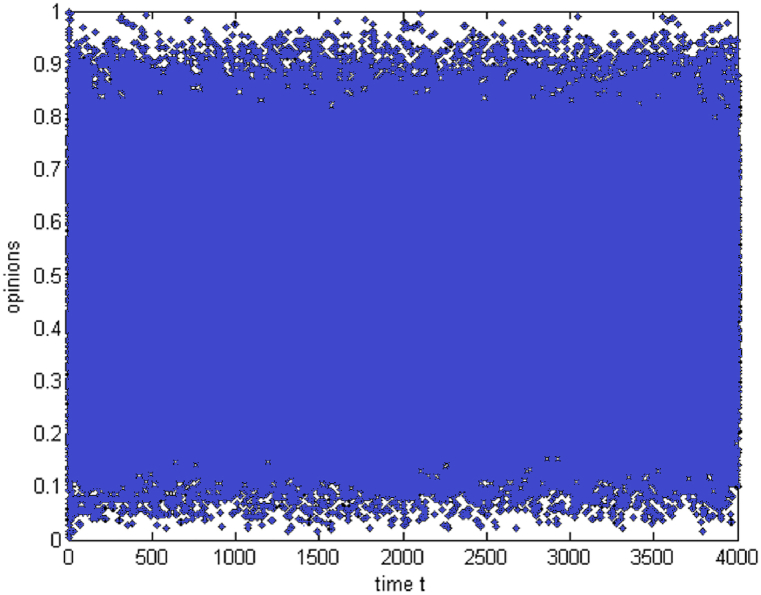
The evolution of group sub-opinions after adding sponsor information from the perspective of two-dimensional opinion.

In group 3, sponsor information is added, and a simulation experiment is performed according to the interaction rules between the sponsor and individual consumers. The results are shown in Fig. 4 below. The sponsor's characteristic values are adjusted, and the influence of two characteristics on group sub-opinions is explored.

Figure (4) highlights neutrality and rupture in opinion dynamics by showing how the addition of a sponsor affects the trend and convergence of group sub-opinions. The simulation results show that the sponsor impacts the evolution of the group sub-opinions. The sponsor changes the trend of the original sub-opinions, gradually converging to neutrality, which causes the group sub-opinions to rupture. In addition, adjusting the range of the two characteristic values shows that the sponsor's characteristics do not affect the evolution of the group sub-options.

(3) The role of the sponsor in the environment of opposing sub-opinions

**Fig. 5:**
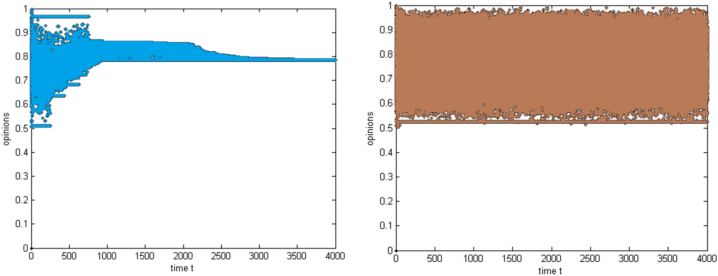
The evolution of group approval sub-opinions before and after sponsor intervention from the perspective of two-dimensional opposing opinions.

**Fig. 6:**
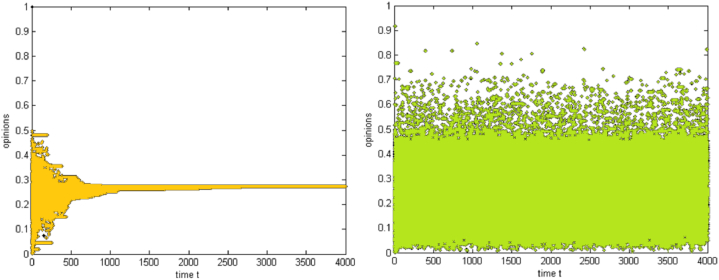
The evolution of group opposition sub-opinions before and after sponsor intervention from the perspective of two-dimensional opposing opinions.

The above simulation experiments show that the threshold and sponsor characteristics do not affect the evolution of group sub-opinion. Then, the initial values are adjusted; the sub-opinion E_j1_ is assumed to have a continuous value between [0.5, 1], and the sub-opinion E_j2_ is supposed to have a continuous value between [0, 0.5]. Next, simulation experiments are performed to explore the sponsor's influence on the evolution of group sub-opinions when sub-opinions are in the opposite position. The simulation results are shown in Fig. 5, Fig. 6.

Figure (5) shows the development of favourable sub-opinions, illustrating the disruption brought on by sponsor action as well as the trend toward consistency and convergence around 0.78 in the absence of a sponsor. Figure (6) illustrates the development of competing sub-opinions, emphasizing how, in the absence of a sponsor, they converge around 0.27 and how sponsor action causes opinion ruptures. The simulation results show that in the absence of a sponsor, the group sub-opinions with a favourable attitude tend to be consistent and converge to 0.78, while the group subopinions with an unfavourable attitude tend to be consistent and converge to 0.27, which are almost in the middle of the range of approval or opposition. After sponsor information is added, group sub-opinions with favourable attitudes evolve and appear to be ruptured, group sub-opinions with opposing attitudes evolve, a few individuals change their opinions, and the opinions of most individuals seem to be ruptured.

The findings are consistent with the body of research on opinion dynamics in social contexts. The study reveals that while sophisticated group structures (Group 4) struggle with opinion fragmentation, smaller group structures (Group 3) typically achieve higher consensus and successful collaborative shopping. This is consistent with previous studies. This is consistent with previous research on the effects of threshold differences and group hierarchy on opinion convergence. Furthermore, recent research indicating that sponsors can affect opinion direction but not significantly alter it with their features alone is corroborated by the modest effect of sponsor characteristics on opinion evolution.

## 5 Discussion

The study's findings shed light on the intricate dynamics of collaborative shopping, providing valuable insights for organizations seeking to exploit group communication in s-commerce. Notably, the study emphasizes the importance of group structure in determining the success of collaborative shopping experiences. Groups with a simple hierarchy, such as Group 3, have a greater chance of success due to shared viewpoints and effective communication routes. In contrast, groups with complex hierarchies, such as Group 4, have difficulty reaching consensus, impeding the success of collaborative shopping projects.

Sponsors play an important role in forming group ideas and driving collaborative shopping activities. While sponsors can affect the direction of group opinions, their attributes, such as professionalism and involvement, have a limited impact on how opinions evolve. Nonetheless, creating effective sponsorship methods, such as selecting sponsors with relevant expertise and developing interactive relationships with customers, is critical for delivering successful collaborative shopping campaigns.

Furthermore, the study emphasizes the role of opinion leaders in fostering collaborative shopping initiatives. These influential individuals can impact group dynamics and play a critical role in influencing consumer behaviour. Businesses may increase their reach and consumer participation by identifying and developing opinion leaders who share their collaborative shopping goals.

Businesses can use these data to optimize collaborative shopping strategies. Selecting appropriate group structures, creating successful sponsorship, and developing opinion leaders are critical elements in enhancing the effectiveness of collaborative shopping projects. By understanding group communication patterns and utilizing the impact of key stakeholders, businesses can increase their competitiveness in the e-commerce landscape.

To assign weights to the elements impacting the influence functions, the analytic hierarchy process mostly depends on expert knowledge. Experts in social dynamics, digital marketing, and consumer behavior were engaged to get insight into how characteristics such as cohesion, conformity, opinion quality, and interactivity impact cooperative shopping practices. A review was conducted of pertinent scholarly works and investigations pertaining to opinion dynamics, group decision-making, and social commerce. These resources provide theoretical frameworks and empirical data that aided in defining the boundaries and relationships among the judgment matrices. Distributed to participants in collaborative shopping platforms were surveys and questionnaires. From the viewpoint of actual users, the responses gave direct insights into the significance of numerous traits (such sponsor professionalism, opinion leader activity, and individual consumer trust tendency). The judgment matrices were refined using data from simulation experiments that simulate various group formations and interaction settings. Researchers might modify the weightings in the matrices to more accurately reflect realistic behaviors by keeping an eye on the results of these simulations.

The purpose of the judgment matrices was to make it easier to apply the AHP to evaluate the relative significance of the variables affecting the convergence coefficient (mu), which is now shown as an influence function. These matrices offer an organized method for evaluating the relative importance of various traits, guaranteeing that the resulting model faithfully captures the intricate interaction between social and individual factors in cooperative shopping.

The matrices ensure a thorough and fair depiction of the factors that drive opinion dynamics in collaborative shopping settings by including a variety of data sources. The suggested impact functions are more robust and applicable because of this multidimensional approach's ability to capture the subtleties of real-world interactions.

## 6 Conclusions and application

### 6.1 Conclusions

This work analyzes collaborative shopping by improving the DW model and developing one-dimensional and two-dimensional opinion interaction rules. The key findings include, that the success of collaborative shopping is largely related to group organization. Group 3, in which all individuals have positive attitudes about the activity, is the most successful. Group 4 is the least successful, with fractured opinions and numerous opponents. Simple group structures tend to promote successful collaborative shopping more effectively than complex ones.

Sponsors can positively influence group opinions about successful collaborative shopping, but they have no substantial effect on the average opinion or promote polarization. The sponsor's attributes, such as professionalism and involvement, have little impact. Two-dimensional opinion dynamics demonstrate that threshold values do not affect the convergence of sub-opinions, which typically settle in an average period. Sponsors can disrupt the convergence but not change the outcome.

These findings indicate that successful collaborative shopping is dependent on group cohesion and basic hierarchies. To improve collaboration, practitioners should focus on developing positive group dynamics and simplifying hierarchical structures. Theoretical consequences include a better understanding of opinion dynamics and sponsorship influence.

The study's simulations may not fully replicate real-world complications, and the role of sponsor characteristics merits more investigation. Future studies should look into real-world applications of these findings, investigate the impact of more diversified sponsor traits, and conduct in-depth analyses of complex hierarchical structures.

### 6.2 Application

Consumers have welcomed collaborative shopping, and it has developed rapidly because of its precise product positioning, satisfactory interactive platform, and price advantage, so understanding the evolution laws of group opinions is the focus of merchants’ attention. The research conclusions have implications for business circles as follows:

A suitable group is selected. Group 3 is most likely to carry out collaborative shopping; for example, on the live-streaming e-commerce hosts the sponsor is also the group manager, who can guide and control the group hierarchy to avoid the adverse impacts of opinion leaders. At the same time, consumers with different backgrounds can be attracted to join an in-group in various ways.

Pay attention to the role of the sponsor. On the one hand, the sponsor selected by the business should have the following characteristics: successful experience in carrying out collaborative shopping or participation in collaborative shopping many times, communication with other consumers enjoyably, entertaining means of expression, etc.; on the other hand, the sponsor should constantly improve his professionalism, sum up experience, find group law, understand group members’ characteristics, hobbies and habits, etc., select product members to demand strongly, at the same time, ensure his interactivity, solve consumers' problems patiently, accept reasonable suggestions gladly, deal with emergencies and after-sales calmly, establish a harmonious interactive relationship with consumers, and coordinate the relationship between businesses and consumers.

In the cultivation of opinion leaders, the fans of opinion leaders are often not influenced by sponsors and tightly gather around them. It is difficult for sponsors to guide the opinions of opinion leaders and their fans. Therefore, the precondition for the smooth development of activities is to cultivate opinion leaders. For opinion leaders who do not support collaborative shopping, it is necessary to listen to reasons in time, improve actively, and try to drag opinion leaders who hold opposing opinions into the supportive camp. In addition, the sponsor can observe the performance of consumers in the group. Consumers who support collaborative shopping, have successful experiences, and have good relationships with members can be cultivated to become opinion leaders and effectively guide the positive evolution of group opinions.

### 6.3 Theoretical implication

The study's findings help to advance the theoretical understanding of collaborative shopping and group dynamics in s-commerce. This study provides a comprehensive framework for analysing group opinions and interaction dynamics in collaborative shopping environments by expanding the dual-process model and incorporating insights from related consumer behavior theories, such as the theory of planned behavior and social identity theory. Furthermore, investigating one-dimensional and two-dimensional opinion interaction rules reveals important insights into the complexities of opinion formation processes.

Overall, this study's theoretical implications show the interdisciplinary nature of s-commerce research, emphasizing the importance of combining multiple theoretical views to understand complex phenomena. Further research could delve deeper into the interaction of group dynamics, individual preferences, and external influences in collaborative purchasing situations, resulting in more nuanced knowledge of consumer behaviour in online group settings.

## Data availability statement

The relevant data for this work are available to the extent reasonably requested by contacting the corresponding author.

## CRediT authorship contribution statement

**Shulin Liang:** Writing – original draft, Software, Resources, Data curation, Conceptualization. **Wang Hu:** Writing – review & editing, Visualization, Validation, Methodology, Investigation.

## Declaration of competing interest

The authors declare the following financial interests/personal relationships which may be considered as potential competing interests:Shulin Liang reports financial support was provided by the General Project of Hunan Social Science Achievements Review Committee in 2023 (XSP2023GLC117). If there are other authors, they declare that they have no known competing financial interests or personal relationships that could have appeared to influence the work reported in this paper.
